# Long-term cardiac outcomes of depression screening, diagnosis and treatment in patients with acute coronary syndrome: the DEPACS study

**DOI:** 10.1017/S003329171900388X

**Published:** 2021-04

**Authors:** Jae-Min Kim, Robert Stewart, Hee-Ju Kang, Seon-Young Kim, Ju-Wan Kim, Hee-Joon Lee, Ju-Yeon Lee, Sung-Wan Kim, Il-Seon Shin, Min-Chul Kim, Hee-Young Shin, Young Joon Hong, Youngkeun Ahn, Myung Ho Jeong, Jin-Sang Yoon

**Affiliations:** 1 Department of Psychiatry, Chonnam National University Medical School, Gwangju, Korea; 2 Psychology and Neuroscience, King's College London, Institute of Psychiatry, London, UK; 3 South London and Maudsley NHS Foundation Trust, London, UK; 4 Department of Cardiology, Chonnam National University Medical School, Gwangju, Korea; 5 Department of Biomedical Science, Chonnam National University Medical School, Gwangju, Korea

**Keywords:** Acute coronary syndrome, cardiac outcome, depression, screening, treatment

## Abstract

**Background:**

To investigate the impacts of depression screening, diagnosis and treatment on major adverse cardiac events (MACEs) in acute coronary syndrome (ACS).

**Methods:**

Prospective cohort study including a nested 24-week randomised clinical trial for treating depression was performed with 5–12 years after the index ACS. A total of 1152 patients recently hospitalised with ACS were recruited from 2006 to 2012, and were divided by depression screening and diagnosis at baseline and 24-week treatment allocation into five groups: 651 screening negative (N), 55 screening positive but no depressive disorder (S), 149 depressive disorder randomised to escitalopram (E), 151 depressive disorder randomised to placebo (P) and 146 depressive disorder receiving medical treatment only (M).

**Results:**

Cumulative MACE incidences over a median 8.4-year follow-up period were 29.6% in N, 43.6% in S, 40.9% in E, 53.6% in P and 59.6% in M. Compared to N, screening positive was associated with higher incidence of MACE [adjusted hazards ratio 2.15 (95% confidence interval 1.63–2.83)]. No differences were found between screening positive with and without a formal depressive disorder diagnosis. Of those screening positive, E was associated with a lower incidence of MACE than P and M. M had the worst outcomes even compared to P, despite significantly milder depressive symptoms at baseline.

**Conclusions:**

Routine depression screening in patients with recent ACS and subsequent appropriate treatment of depression could improve long-term cardiac outcomes.

## Introduction

Depression is common in acute coronary syndrome (ACS) including myocardial infarction (MI) and unstable angina. Comorbid depression has been robustly associated with poor prognosis of ACS including increased mortality and non-fatal events (Nicholson, Kuper, & Hemingway, 2006). Since ACS and depression are two leading causes of disability (Mathers, Fat, & Boerma, 2008), their comorbidity may generate a high disease burden. Accordingly, the screening and treatment of depression have been considered as potentially important, although there has been no consensus on applying this procedure to real clinical practice for patients with ACS.

In 2008, the American Heart Association (AHA) Science Advisory recommended routine screening for depression in patients with ACS considering the deleterious effects of depression on ACS prognosis (Lichtman et al., 2008). However, shortly afterward, a systematic review found no evidence for or against the recommendations that depression should be evaluated or that screening for depression should be considered as part of standard care in patients with ACS (Thombs et al., 2008). This argument was strongly influenced by the lack of evidence for significant beneficial effects of antidepressant or cognitive behavioural treatment for depression on long-term cardiac outcomes in patients with ACS (Berkman et al., 2003; Glassman, Bigger, & Gaffney, 2009; van Melle et al., 2007). On the one hand, the need for appropriate screening guidelines has been highlighted because depression is an important cardiac risk marker, is a treatable condition, and its presence warrants more aggressive cardiac care and secondary prevention efforts regardless of whether treating depression can improve cardiac outcomes (Carney, Freedland, & Jaffe, 2009). On the other hand, there have been additional calls for reassessing AHA recommendations for routine screening of depression because of the extra time and cost involved (Thombs et al., 2013; Ziegelstein, Thombs, Coyne, & de Jonge, 2009), particularly given the pressing need for treatment of ACS in its acute phase (Anderson et al., 2013), and the high costs associated with ACS (Benjamin et al., 2018).

Recently evidence has emerged on potential beneficial effects of treating depression on cardiac prognosis in ACS. In the TRIUMPH study (Translational Research Investigating Underlying Disparities in Acute Myocardial Infarction Patients' Health Status), the 1-year mortality was higher in patients with untreated depression and not raised in patients with treated depression compared to those without depression (Smolderen et al., 2017). Our DEPACS (DEPression in Acute Coronary Syndrome) study group reported findings from a randomised clinical trial (RCT), in which 24-week treatment with escitalopram compared with placebo was associated with a lower risk of major adverse cardiac event (MACE) after a median 8.4 years follow-up in patients with depression following recent ACS (Kim et al., 2018*c*). By extending this analysed cohort, we aimed to investigate more comprehensively whether depression screening, further diagnosis, and subsequent treatment had differential associations with longer-term cardiac outcomes following ACS. Other psychiatric diagnoses or problems such as anxiety and suicidal ideation have also been associated with cardiac outcomes and are commonly comorbid with depression. We have published these issues in other publications (Kim et al., 2018*a*, 2018*b*), hence we focused on depression issue here to address the evidence gap in this area.

## Methods

### Study outline and participants

**Fig. 1.**
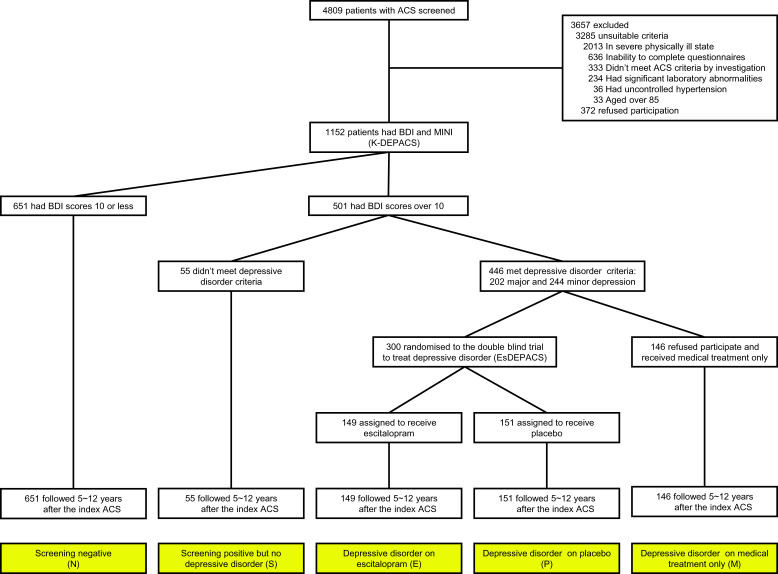
Study outline and participants recruitment process. ACS, acute coronary syndrome; K-DEPACS, Korean DEPression in Acute Coronary Syndrome study; BDI, Beck Depression Inventory; DSM-IV, Diagnostic and Statistical Manual of Mental Disorders, fourth edition; EsDEPACS, Escitalopram for DEPression in Acute Coronary Syndrome study.

The analyses described in this study were carried out *post-hoc* using data from a prospective observational study of patients with ACS, Korean DEPACS (K-DEPACS), which also included an RCT for patients with depressive disorder and ACS: Escitalopram for DEPACS (EsDEPACS). The design and main findings of K-DEPACS and EsDEPACS have been published (Kim et al., 2018*c*, 2018*a*), and the eligibility criteria are described in the online Supplementary material. Written informed consent was collected for both studies, which were approved by the Chonnam National University Hospital (CNUH) Institutional Review Board. The outline and participant recruitment process for analysis performed in this study are presented in Fig. 1.

### K-DEPACS baseline evaluation

From 2006 to 2012, the K-DEPACS participants were consecutively recruited from patients recently hospitalised with ACS (*N* = 4809) at the Department of Cardiology of CNUH, Gwangju, South Korea. Patients were treated by the study cardiologists based on international guidelines for the management of ACS (Anderson et al., 2013). Those who met eligibility criteria and agreed to participate comprised the K-DEPACS sample (*N* = 1152), and were screened for depressive symptoms with the Beck Depression Inventory (BDI) (Beck, Ward, Mendelson, Mock, & Erbaugh, 1961) at baseline as inpatients within 2 weeks (mean 6.3 ± 2.4 days) post-ACS and thereafter as outpatients every 4 weeks up to 12 weeks. The BDI was chosen as a depression screen to retain consistency with a previous randomised controlled trial for treating depression in ACS (Glassman et al., 2002). Those screening positive (BDI>10, *N* = 501) at any of these occasions received a clinical evaluation by the study psychiatrists using the Mini-International Neuropsychiatric Interview (MINI) (Sheehan et al., 1998), a structured diagnostic psychiatric interview for Diagnostic and Statistical Manual of Mental Disorders, Fourth Edition (DSM-IV) (American Psychiatric Association, 1994), defining unipolar major or minor depressive disorder categories as outputs.

Information was collected regarding characteristics that could potentially affect cardiac outcomes (Jaffe et al., 2006; Panteghini, 2004). Demographic data were obtained on age, sex, education, marital status, living alone, housing and employment status. Concerning psychiatric characteristics, the scores on Hamilton Rating Scale for Depression (HAMD) (Hamilton, 1960) and Hospital Anxiety and Depression Scale depression anxiety subscale (HADS-A) (Bjelland, Dahl, Haug, & Neckelmann, 2002), and previous and family history of depression were recorded. The following cardiovascular risk factors were ascertained: diagnosed hypertension and diabetes mellitus, hypercholesterolemia by fasting serum total cholesterol level (>200 mg/dL), obesity (based on measured body mass index), reported current smoking status and previous and family history of ACS. Cardiac severity status was also characterised by Killip classification (Killip & Kimball, 1967), left ventricular ejection fraction and serum levels of troponin I and creatine kinase-MB. Frequencies of the classes of cardiovascular medications used were recorded: calcium channel blockers, nitrates, *β*-blockers, angiotensin-converting enzyme inhibitors, angiotensin 2 receptor blocker, statins, aspirin, antiplatelets and diuretics.

### The nested randomised controlled trial: EsDEPACS study

Of the 501 screen-positive participants, 55 had no depressive disorder. Of the remaining 446 patients with a diagnosis of major (*N* = 202) or minor (*N* = 244) depressive disorder, 300 who met the eligibility criteria and agreed to participate were enrolled in a 24-week, double-blind, placebo-controlled RCT of escitalopram, the EsDEPACS study (ClinicalTrial.gov registry number: NCT00419471). Higher participation rates were noted in those with more severe depressive symptoms, resulting in major depressive disorder prevalences of 57.0% (85/149) in escitalopram-allocated and 55.6% (84/151) in placebo-allocated participants, compared to 22.6% (33/146) in the remaining patients with the depressive disorder who were not randomised. The first patient was enrolled in May 2007, and the last patient completed follow-up evaluation in March 2013. Examinations were scheduled at baseline, and in weeks 4, 8, 12, 16, 20 and 24 thereafter. The dose of the study drug was 10 mg/day initially and could be changed (from 5 to 20 mg/day) according to the investigators' clinical decision, taking into account response and tolerability after the second evaluation. The mean (s.d.) doses at the last visit were 7.6 (3.7) mg for the escitalopram group and 8.5 (3.9) mg for the placebo group. Adherence was checked by pill counts at every visit, and was defined as acceptable if at least 75%. Adherence to medications was 93.3% and 95.4% in patients receiving escitalopram and placebo, respectively. Depression treatment, including antidepressant use other than study drugs, was not allowed during the study period. With respect to the ethics of the use of placebo in patients with depressive symptoms, the following considerations should be noted: (i) because of the lack of evidence for the effect of depression treatment shortly following ACS at the time the study was designed, the EsDEPACS trial was judged both by funders and an independent ethics review panel to be addressing an issue of clinical equipoise (Kim et al., 2015*a*); (ii) besides providing study drugs, research psychiatrists met with the patients for at least 30 min at every visit and evaluated their psychological symptoms using simple support and reassurance after cardiology treatment; (iii) participants could withdraw from the trial at any point and for any reason; (iv) for participants without remission after the trial, further treatment was facilitated when requested; (v) all participants were approached for the evaluation of psychiatric outcomes 1 year after baseline evaluation (Kim et al., 2015*b*). The details and main results of this trial have been published previously (Kim et al., 2015*a*), in which escitalopram was superior to placebo in treating depression without significant difference in adverse events. The remaining 146 participants who did not meet the eligibility criteria or declined participation in the trial received conventional medical treatment for ACS only (MTO). The MTO and screen-positive but had no depressive disorder participants were recommended treating depressive symptoms wherever possible.

### Long-term cardiac outcomes

Comprehensive evaluations for cardiac outcomes were conducted for data on hospital admissions, deaths, recurrent MI and percutaneous coronary intervention (PCI). To enable non-hierarchic endpoint analyses, all patients were followed for the evaluation point of interest or until death. The primary endpoint was a MACE, which was a composite of all-cause mortality, MI and PCI (excluding non-emergent PCIs). Secondary endpoints were all-cause mortality, cardiac death (defined as sudden death when no other explanation was available, death from arrhythmias or after MI or heart failure, or death caused by heart surgery or endocarditis), MI and PCI. An independent endpoint committee composed of study cardiologists adjudicated all potential events, blind to participants' depression status.

### Statistical analysis

According to the depression screening, diagnosis and treatment status at baseline, participants were divided into five groups: 651 screening negative, 55 screening positive but not found to have a depressive disorder, 149 with depressive disorder randomised to escitalopram, 151 with depressive disorder randomised to placebo and 146 with depressive disorder receiving MTO. Demographic and clinical characteristics at baseline were compared between the five groups using analysis of variance or χ^2^ tests with *post-hoc* Scheffe's tests, or using individual pairwise *post-hoc* comparisons between the five groups as appropriate. Characteristics significantly associated with group status (*p* < 0.05) and/or with potential effects on MACE were used as covariates *a priori* in further adjusted analyses. For investigating the effects of depression screening and diagnosis on long-term MACE in ACS, Kaplan–Meier curves were constructed and the cumulative proportion of MACE by negative *v.* positive screening status (and further by screening positive with *v.* without depressive disorder status) at baseline was compared using the log-rank tests. Cox proportional hazards models were used to assess the time to MACE after adjustment for the potential covariates described above. For investigating subsequent depression treatment effects according to treatment allocations, the same Cox proportional hazards models were used among the four depressive screening-positive groups with individual pairwise *post-hoc* comparisons. Sensitivity analyses were carried out excluding patients taking antidepressants at the 1-year post-ACS examination to exclude the possible medication effects on long-term cardiac outcomes. To adjust for an overall type I error rate of *p* < 0.05 for multiple comparisons, Bonferroni corrections were conducted (five comparisons: a primary and four secondary endpoints; 0.05/5 = 0.01) in the long-term cardiac outcome analyses. Statistical analyses were carried out using SPSS 21.0 software.

## Results

### Baseline characteristics for the five comparison groups

Baseline characteristics between the five groups are compared in Table 1. Significant group differences were found in age, sex, education, marital status, living alone, accommodation, employment status, scores on HAMD and HADS-A, hypertension, diabetes, smoking status and family history of ACS (all *p* < 0.05). Considering these results and previous research (Bjelland et al., 2002; Killip and Kimball, 1967), 10 variables (see footnotes of Tables 2 and 3) were included as covariates in subsequent analyses. Results of *post-hoc* comparisons are summarised in the last column of Table 1. In particular, the mean scores on HAMD and HADS-A in the ‘depressive disorder on MTO’ group were significantly lower than in those with depressive disorder receiving escitalopram or placebo, but were similar to those screening positive but with no depressive disorder diagnosis.

**Table 1.** Baseline characteristics by depression screening, diagnosis and treatment status in 1152 patients with acute coronary syndrome (ACS)

	Screening negative (N) (*N* = 651)	Screening positive but no depressive disorder (S) (*N* = 55)	Depressive disorder on escitalopram (E) (*N* = 149)	Depressive disorder on placebo (P) (*N* = 151)	Depressive disorder on medical treatment only (M) (*N* = 146)	Statistics for group differences^a^	*Post-hoc* comparisons (*p* < 0.05)
Demographic characteristics							
Age, mean (s.d.) year	58.3 (11.5)	55.0 (9.4)	60.0 (11.2)	60.1 (10.5)	58.3 (11.6)	*F* = 2.753 *p* = 0.027	n-s
Sex, N (%) men	514 (79.0)	44 (80.0)	89 (59.7)	93 (61.6)	84 (57.5)	χ^2^ = 51.116 *p* < 0.001	N,S > E,P,M
Education, mean (s.d.) year	10.2 (4.8)	9.4 (4.4)	9.4 (4.2)	9.2 (4.1)	9.0 (5.1)	*F* = 3.166 *p* = 0.013	n-s
Unmarried marital status, N (%)	84 (12.9)	10 (18.2)	18 (12.1)	29 (19.2)	35 (24.0)	χ^2^ = 14.698 *p* = 0.005	N,E < M
Living alone, N (%)	54 (8.3)	6 (10.9)	12 (8.1)	16 (10.6)	24 (16.4)	χ^2^ = 9.706 *p* = 0.046	N,E < M
Rented accommodation, N (%)	73 (11.2)	13 (23.6)	21 (14.1)	34 (22.5)	28 (19.2)	χ^2^ = 19.582 *p* = 0.001	N < S,P,M
Unemployed status, N (%)	217 (33.3)	22 (40.0)	71 (47.7)	73 (48.3)	67 (45.9)	χ^2^ = 21.939 *p* < 0.001	N < E,P,M
Psychiatric characteristics							
HAMD, mean (s.d.) score	3.2 (2.3)	7.6 (3.1)	15.9 (4.8)	15.6 (4.8)	10.9 (3.4)	*F* = 783.607 *p* < 0.001	N < S,M < E,P
HADS-A, mean (s.d.) score	3.5 (1.2)	4.9 (1.2)	6.3 (2.5)	6.2 (2.4)	4.3 (1.89)	χ^2^ = 136.231 *p* < 0.001	N < S,M < E,P
Previous depression, N (%)	20 (3.1)	1 (1.8)	6 (4.0)	7 (4.6)	8 (5.5)	χ^2^ = 3.013 *p* = 0.556	n-s
Family history of depression, N (%)	16 (2.5)	2 (3.6)	5 (3.4)	6 (4.0)	4 (2.7)	χ^2^ = 1.310 *p* = 0.860	n-s
Cardiac risk factors, N (%)							
Hypertension	283 (43.5)	21 (38.2)	90 (60.4)	94 (62.3)	68 (46.6)	χ^2^ = 29.018 *p* < 0.001	N,S,M < E,P
Diabetes mellitus	116 (17.8)	6 (10.9)	44 (29.5)	41 (27.2)	28 (19.2)	χ^2^ = 17.746 *p* = 0.001	N,S < E,P M < E
Hypercholesterolemia	324 (49.8)	26 (47.3)	73 (49.0)	71 (47.0)	78 (53.4)	χ^2^ = 1.404 *p* = 0.844	n-s
Obesity	283 (43.5)	23 (41.8)	59 (39.6)	65 (43.0)	60 (41.1)	χ^2^ = 0.911 *p* = 0.923	n-s
Current smoker	261 (40.1)	26 (47.3)	44 (29.5)	42 (27.8)	44 (30.1)	χ^2^ = 16.981 *p* = 0.002	N,S > E,P,M
Previous ACS	19 (2.9)	4 (7.3)	8 (5.4)	11 (7.3)	8 (5.5)	χ^2^ = 8.298 *p* = 0.081	n-s
Family history of ACS	11 (1.7)	6 (10.9)	9 (6.0)	8 (5.3)	5 (3.4)	χ^2^ = 20.142 *p* < 0.001	N < S,E,P S > M
Current cardiac status							
Killip class >1, N (%)	114 (17.5)	5 (9.1)	24 (16.1)	35 (23.2)	28 (19.2)	χ^2^ = 6.327 *p* = 0.176	n-s
LVEF, mean (s.d.) percent	61.7 (11.0)	58.2 (13.8)	60.4 (11.0)	61.9 (10.4)	60.1 (12.6)	*F* = 1.983 *p* = 0.095	n-s
Troponin I, mean (s.d.) mg/dL	9.4 (16.0)	10.2 (17.9)	9.7 (8.5)	9.7 (8.0)	9.6 (16.0)	*F* = 0.060 *p* = 0.993	n-s
CK-MB, mean (s.d.) mg/dL	16.4 (39.5)	23.9 (54.6)	17.2 (22.0)	16.3 (20.8)	18.6 (42.3)	*F* = 0.607 *p* = 0.658	n-s
Cardiovascular medications							
Calcium channel blockers	204 (31.3)	18 (32.7)	51 (34.2)	59 (39.1)	45 (30.8)	χ^2^ = 1.829 *p* = 0.767	n-s
Nitrates	504 (77.4)	42 (76.4)	116 (77.9)	114 (75.5)	102 (69.9)	χ^2^ = 0.560 *p* = 0.967	n-s
*β*-blockers	471 (72.4)	42 (76.4)	108 (72.5)	106 (70.2)	109 (74.7)	χ^2^ = 0.183 *p* = 0.996	n-s
Angiotensin-converting enzyme inhibitors	201 (30.9)	18 (32.7)	45 (30.2)	51 (33.8)	48 (32.9)	χ^2^ = 0.394 *p* = 0.983	n-s
Angiotensin 2 receptor blocker	329 (50.4)	27 (49.1)	76 (51.0)	76 (50.3)	74 (50.7)	χ^2^ = 0.021 *p* = 0.999	n-s
Statins	482 (74.0)	41 (74.5)	119 (79.9)	116 (76.8)	108 (74.0)	χ^2^ = 0.355 *p* = 0.986	n-s
Aspirin	603 (92.7)	51 (92.7)	132 (88.6)	136 (90.1)	135 (92.5)	χ^2^ = 0.146 *p* = 0.997	n-s
Antiplatelets	502 (77.1)	45 (81.8)	113 (75.8)	113 (74.8)	112 (76.7)	χ^2^ = 0.158 *p* = 0.997	n-s
Diuretics	123 (18.9)	11 (20.0)	32 (21.5)	29 (19.2)	25 (17.1)	χ^2^ = 0.650 *p* = 0.957	n-s

HAMD, Hamilton Rating Scale for Depression; HADS-A, Hospital Anxiety and Depression Scale anxiety subscale; LVEF, left ventricular ejection fraction; CK-MB, creatine kinase-MB.

a Analysis of variance or χ^2^ tests as appropriate.

**Table 2.** Effects of depression screening and diagnosis status on long-term cardiac outcomes in 1152 patients with acute coronary syndrome (ACS)

Cardiac outcomes	Group by depression screening and diagnosis status	*N* patient	*N* (%) event	All participants (*N* = 1152)		Screening positive participants (*N* = 501)	
				AHR (95% CI)^a^	*p*	AHR (95% CI)^a^	*p*
Major adverse cardiac event	Screening negative	651	193 (29.6)	Ref			
	Screening positive	501	253 (50.5)	2.16 (1.64–2.84)	**<0.001**			
	No depressive disorder	55	24 (43.6)	2.04 (1.28–3.27)	**0.003**	Ref	
	Present depressive disorder	446	229 (51.3)	2.18 (1.64–2.88)	**<0.001**	1.06 (0.69–1.65)	0.822
All cause death	Screening negative	651	86 (13.2)	Ref			
	Screening positive	501	125 (25.0)	2.26 (1.52–3.36)	**<0.001**		
	No depressive disorder	55	14 (25.5)	2.85 (1.50–5.40)	**0.001**	Ref	
	Present depressive disorder	446	111 (24.9)	2.18 (1.47–3.27)	**<0.001**	0.79 (0.45–1.44)	0.435
Cardiac death	Screening negative	651	41 (6.3)	Ref			
	Screening positive	501	70 (14.0)	2.31 (1.35–3.96)	**0.002**			
	No depressive disorder	55	9 (16.4)	3.03 (1.35–6.81)	**0.007**	Ref	
	Present depressive disorder	446	61 (13.7)	2.20 (1.29–3.81)	**0.005**	0.77 (0.36–1.62)	0.459
Myocardial infarction	Screening negative	651	47 (7.2)	Ref			
	Screening positive	501	63 (12.6)	1.59 (0.90–2.79)	0.108		
	No depressive disorder	55	4 (7.3)	0.95 (0.33–2.88)	0.900	Ref	
	Present depressive disorder	446	59 (13.2)	1.68 (0.95–2.97)	0.081	1.80 (0.63–5.28)	0.282
Percutaneous coronary intervention	Screening negative	651	73 (11.2)	Ref			
	Screening positive	501	89 (17.8)	1.92 (1.23–3.06)	**0.006**		
	No depressive disorder	55	6 (10.9)	1.41 (0.57–3.45)	0.459	Ref	
	Present depressive disorder	446	83 (18.6)	2.01 (1.26–3.24)	**0.003**	1.32 (0.57–3.12)	0.540

a Adjusted hazards ratio (95% confidence interval) [AHR (95% CI)] after adjustment for age, sex, marital status, employment, scores on Hamilton Rating Scale for Depression and Hospital Anxiety and Depression Scale anxiety subscale, hypertension, diabetes, smoking and left ventricular ejection fraction.

Values in bold type represent statistical significance after Bonferroni correction.

**Table 3.** Effects of depression treatment status on long-term cardiac outcomes in 501 patients with acute coronary syndrome (ACS)

Cardiac outcomes, N (%)	Screening positive but no depressive disorder (S) (*N* = 55)	Depressive disorder on escitalopram (E) (*N* = 149)	Depressive disorder on placebo (P) (*N* = 151)	Depressive disorder on medical treatment only (M) (*N* = 146)	Statistics for group differences^a^	*Post-hoc* comparisons (*p* < 0.05)
Major adverse cardiac event	24 (43.6)	61 (40.9)	81 (53.6)	87 (59.6)	Wald = 43.276 *p* < **0.001**	E < P<M
All-cause mortality	14 (25.5)	31 (20.8)	37 (24.5)	43 (29.5)	Wald = 18.157 *p* < **0.001**	S > E E,P < M
Cardiac death	9 (16.4)	16 (10.7)	20 (13.2)	25 (17.1)	Wald = 9.162 *p* = **0.002**	n-s
Myocardial infarction	4 (7.3)	12 (8.1)	12 (15.2)	23 (15.8)	Wald = 8.691 *p* = **0.003**	E < M
Percutaneous coronary intervention	6 (10.9)	19 (12.8)	30 (19.9)	34 (23.3)	Wald = 17.431 *p* < **0.001**	E < P,M

a Cox proportional hazard tests after adjusted age, sex, marital status, employment, scores on Hamilton Rating Scale for Depression and Hospital Anxiety and Depression Scale anxiety subscale, hypertension, diabetes, smoking and left ventricular ejection fraction.

Patients can have more than one event.

Values in bold type represent statistical significance after Bonferroni correction.

### MACE occurrences according to depression screening and diagnosis status

**Fig. 2.**
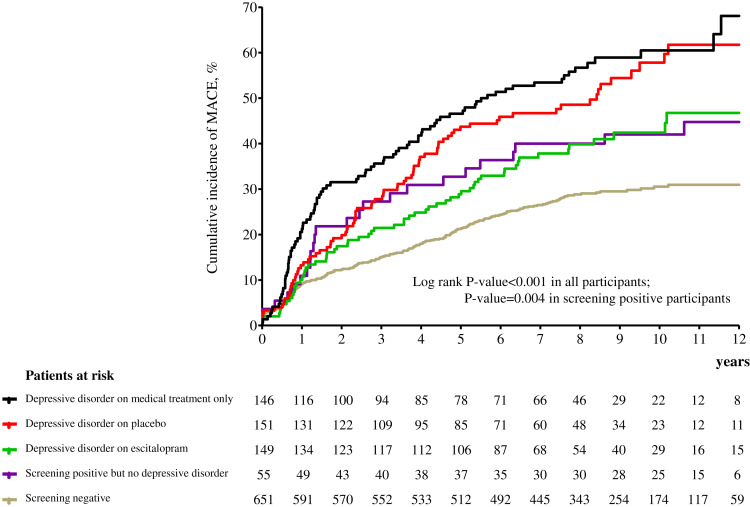
Cumulative incidence (%) of major adverse cardiac events (MACE) by time (years) in 1152 patients with acute coronary syndrome (ACS).

In 2017, all participants were followed for 5–12 years or until they died [median; mean (s.d.) follow-up = 8.4; 8.7 (1.5) years]. The primary endpoint (composite MACE) occurred in 446 (38.7%) participants. Considering secondary endpoints, all-cause mortality occurred in 211 (18.3%), cardiac death in 111 (9.6%), MI in 110 (9.5%) and PCI in 162 (14.1%) participants. Cumulative composite MACE incidences in the five exposure groups are illustrated in Fig. 2 [screening negative 29.6% (193/651), screening positive but no depressive disorder 43.6% (24/55), depressive disorder on escitalopram 40.9% (61/149), depressive disorder on placebo 53.6% (81/151) and depressive disorder on MTO 59.6% (87/146)]. Significant group differences were found across all five exposure categories and between the four depression screen-positive groups. Antidepressants were being taken by 19 participants (eight screening negative, one screening positive but no depressive disorder, five on depressive disorder on escitalopram, three on depressive disorder on placebo and two on depressive disorder on MTO) at the 1-year follow-up point. When the same analyses were repeated after excluding these participants, the results were not changed substantially. The incidences of the composite MACE outcome and its individual components are compared in Table 2 according to depression screen-positive/negative status and between the screen-positive subgroups with/without depressive disorder diagnostic criteria. In the total sample, participants screening positive for depressive disorder had significantly higher hazards of composite and all individual MACE components except for MI compared to those screening negative after full adjustment. Compared to the screen-negative group, those screening positive but not fulfilling diagnostic criteria for depressive disorder retained significantly higher hazards of composite MACE, all-cause mortality and cardiac death; and those screening positive with a depressive disorder diagnosis had significantly higher hazards of composite and all individual MACE components except for MI. However, in the screen-positive group, no significant differences were found in MACE incidences between those with and without diagnostic criteria for depressive disorder.

### MACE occurrences according to subsequent depression treatment status

Comparisons of the four depression screen-positive subgroups after the same adjustments are described in Table 3. Significant group differences in all MACE outcomes were found. In *post-hoc* comparisons (*p* < 0.05), those randomised to escitalopram displayed better outcomes in composite MACE and PCI compared to both those randomised to placebo and those receiving MTO, and lower all-cause mortality and MI compared to those receiving MTO. Participants randomised to placebo had better outcomes in composite MACE and all-cause mortality compared to those receiving MTO. The screen-positive group without diagnostic criteria for the depressive disorder had higher all-cause mortality compared to participants randomised to escitalopram, but did not differ significantly from other depression treatment groups.

## Discussion

In this median 8.4-year follow-up of patients with recent ACS, screening positive for depression on the BDI was associated with worse long-term cardiac outcomes, even in the cases who did not fulfil diagnostic criteria for depressive disorder. Of patients in this screen-positive group, those randomised to 24 weeks of escitalopram treatment experienced better cardiac outcomes than those randomised to placebo and those with the diagnosed depressive disorder who were not randomised and received MTO instead. This latter MTO group had worse outcomes than those receiving placebo, despite having significantly milder depressive symptoms at baseline.

In this study, worse long-term cardiac outcomes in the screen-positive group were largely consistent with previous reports using depressive symptom scales for categorising depression (Lesperance, Frasure-Smith, Talajic, & Bourassa, 2002). A meta-analysis of 26 studies of various heart diseases, cardiac outcomes and follow-up duration, using depressive symptom scales estimated the hazards ratio associated with depressive symptoms to be 1.92 (Nicholson et al., 2006). The equivalent hazards ratio in the cohort described here was 2.15, and thus higher than the meta-analysed pooled result. The cohort in our study was a homogeneous diagnostic group with comprehensive cardiac outcomes and longer follow-up, all of which are recommended in cardiac outcome studies (Galløe et al., 2008).

Through the further application of a diagnostic protocol for depressive disorder, it was possible to discriminate the group who screened positive for depression but did not fulfil diagnostic criteria for depressive disorder. As far as we are aware, long-term cardiac outcomes in this particular group have not previously been evaluated specifically. The strengths of the associations with adverse outcomes were not statistically different in this group compared to those with diagnosed depressive disorder. However, statistically significant associations were found for fatal rather than non-fatal outcomes, partly consistent with a previous research report that even minimal symptoms of depression increase the mortality risk after acute MI (Bush et al., 2001). These results suggest that screening of depression for identifying even minor depressive symptoms might be meaningful in terms of predictive values in patients with ACS. They provide some support for further formal diagnostic assessment for depressive disorder which would be beyond current recommendations of AHA Science Advisory (Lichtman et al., 2008); however, drawing conclusions should be cautious, since there were no direct comparisons between a screened and non-screened group in this study, and the size of this group was small, which may increase the possibility of type II error.

The cumulative incidence of composite MACE was significantly lower in people with depressive disorder randomised to escitalopram group (40.9%) compared both to those randomised to placebo (53.6%) and those not randomised and receiving MTO (59.6%). In this study cohort, we have previously reported that escitalopram treatment for depression after a recently developed ACS was associated with better psychiatric outcomes including depressive symptoms, sleep, social function and quality of life at the 24-week endpoint compared to placebo (Kim et al., 2015*a*, 2015*b*) as well as at 1 year after the index ACS compared to placebo and MTO (Kang et al., 2015). Recently, we also reported that escitalopram treatment was associated with better long-term cardiac outcomes compared with placebo (Kim et al., 2018*c*). Moreover in the present study, those randomised to escitalopram had significantly lower all-cause mortality even compared to the screen-positive group without diagnostic criteria for depressive disorder. All these results suggest that successful treatment of depression and thereby improvement of psychiatric outcomes after the recent ACS could modify the long-term prognosis of ACS. Our findings are supported by previous observations that non-remission of depression is associated with unhealthy behaviour including sedentary lifestyle and treatment non-adherence to cardiovascular drugs, which are predictors of worse ACS prognosis (Whooley et al., 2008). One consideration is that the Enhancing Recovery in Coronary Heart Disease Patients (ENRICHD) trial found the intervention (9 months of cognitive behavioural therapy with or without 12 months of antidepressants) had significant beneficial effects on improving depressive symptoms compared to usual care among 2481patients with depression following MI (Berkman et al., 2003), but nonetheless found no difference in cardiac outcomes over a 29-month follow-up between the two groups (van Melle et al., 2007). A meta-analysis of 16 studies of depression following ACS found that differences in MACE occurrence became prominent over longer follow-up intervals (Meijer et al., 2013); thus our positive findings probably will be due to the longer follow-up period.

An interesting finding is that the depressive disorder on MTO group showed the worst long-term cardiac outcomes, compared not only to the escitalopram but also to the placebo group despite the fact that the group receiving MTO had less severe depressive and anxiety symptoms at baseline (Table 1). This may be due to those with mild depressive symptoms being more likely to refuse to participate. On the other hand, it may be related to generic input provided in the RCT. As stated earlier, the research psychiatrists met the patients allocated to escitalopram or placebo at least 30 min at every visit for evaluating psychological symptoms with simple supports and reassurance after the cardiologists' treatment, while the MTO group was only formally provided with cardiologists' treatment. The input received by both treatment and placebo groups in the RCT may thus have similarities to collaborative care, which has been cited as effective for treating psychological symptoms and for reducing cardiac symptoms during the treatment period (Davidson et al., 2010). The beneficial effect of placebo over MTO may be due to this kind of active psychological care with higher levels of attention as well as the placebo effect itself. Similar findings have been observed in patients with major depressive disorder, in that treatment enrolment with pill-taking, regardless of whether this involves the active drug or placebo, promotes therapeutic alliance and increased response compared to conventional treatment (Leuchter, Hunter, Tartter, & Cook, 2014). Related to this, the difference in MACE between the groups was driven largely by PCI, suggesting that patients closer to therapy might have interacted in a more consistent basis with physicians, thus potentially accounting for the better outcomes.

Based on our findings, several suggestions can be proposed. First, routine depression screening is recommended for identifying a group at risk of long-term adverse cardiac outcomes (approximately two times higher based on previous findings and our own) in all recently developed ACS patients. Although direct ACS treatment is urgent, intensive and required to prevent early mortality, there remains potential time to administer simple depression screening scales following initial stabilisation and before hospital discharge. Second, depression treatment with antidepressants, particularly with escitalopram (Kim et al., 2015*a*), can be proposed for patients with depressive disorders. The treatment may reduce the depressive symptoms as well as improve the long-term cardiac outcomes (Kim et al., 2018*c*). Third, for patients with depressive disorders but who do not want to take antidepressants, collaborative care with psychiatric personnel could be considered. Since integrated collaborative care has been shown to be effective for reducing both depressive and cardiac symptoms during the treatment period (Meijer et al., 2013), long-term follow-up study results are anticipated. Fourth, for those who have depressive symptoms but do not accept any psychiatric help, more careful monitoring with regular examinations for cardiovascular status might reduce the occurrence of MACE (Carney et al., 2009), while these need further investigation.

**Fig. 3.**
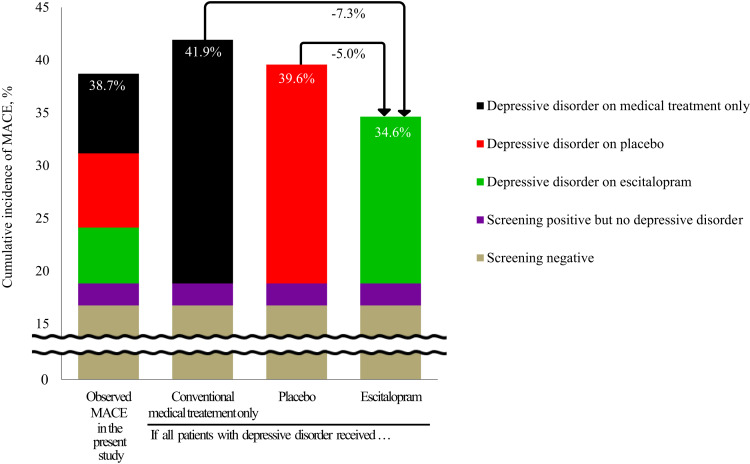
Approximated cumulative incidence (%) of major adverse cardiac events (MACE) by depression treatment status in patients with acute coronary syndrome (ACS). If the depression screening–diagnosis–treatment package were to be administered to a hypothetical 1000 patients with recent ACS, the number of MACE instances over a median 8.4-year follow-up period could be reduced by 73 or 50 compared respectively to medical treatment only or placebo-equivalent conditions.

Effectiveness of long-term cardiac outcomes can be approximated from the present findings, summarised visually in Fig. 3. Composite MACE occurred in 446 (38.7%) of 1152 participants with ACS during a median 8.4-year follow-up. After depression screening and diagnosis, 446 patients were diagnosed as having depressive disorders. If all these patients with depressive disorders received just conventional MTO, the number of MACE expected in all participants would be 483 [41.9%; 193 in those screening negative, plus 24 in those screening positive but with no depressive disorder, plus 266 (59.6%) in the 446 with depressive disorder]. More conservatively, if it is assumed that all patients with depressive disorder received placebo, the number of MACE expected in all participants would be 456 [39.6%; 193 in those screening negative, plus 24 in those screening positive but with no depressive disorder, plus 239 (53.6%) in the 446 with depressive disorder]. However, if all these patients received escitalopram, the number of MACE expected in all participants would be 399 [34.6%; 193 in those screening negative, plus 24 in those screening positive but with no depressive disorder, plus 182 (40.9%) in the 446 with depressive disorder]. Summing up, if the depression screening–diagnosis–treatment package were to be administered to a hypothetical 1000 patients with recent ACS, the number of MACE instances over a 5–12-year period (i.e. that covered in our study) could be reduced by 73 or 50 compared, respectively, to MTO- or placebo-equivalent conditions. Additionally, the effects of interventions for depressive symptoms on long-term cardiac outcomes in the screen-positive group without diagnostic criteria for depressive disorder have not been evaluated yet, although would be worth investigating considering the negative impact of depressive symptoms reported by previous research and our own study.

Limitations should also be considered. First, recruitment was carried out at a single site, which may limit the generalisability of the present findings, although a single-centre study has potential strengths in terms of consistency in evaluation and treatment. Second, the MTO group was not randomly assigned but was formed of those patients with depressive disorder who refused or were non-eligible to take part in the clinical trial, since the present analyses were not pre-planned. This group is thus not equivalent at baseline to the randomised groups and subject to selection bias; specifically, the baseline features of the MTO group indicate lower depression and anxiety symptoms. However, this bias may obscure rather than magnify the findings, and all data on longitudinal effects were adjusted for baseline scores on depression and anxiety symptoms. Future studies are needed with randomisation or other means to evaluate (e.g. by propensity-matching) MTO as an intervention to clarify this issue. Third, antidepressants use after the RCT was investigated only one time at the 1-year follow-up point, although the number was small (19 participants). Related to this, no attempt was made to investigate the effect of non-pharmacologic treatment for depression or other mental health conditions during the follow-up period, although within the Korean healthcare system this would be relatively uncommon.

## Conclusion

Depressive disorder can be reasonably identified by routine screening in all patients with recent ACS and subsequent appropriate treatment should be recommended to those who screen positive and meet diagnostic criteria, which could improve long-term cardiac outcomes. Considering service impact, the routine administration of depression screening scales need not require lengthy or expensive assessments, although further treatment of depression does indeed give rise to potential time and cost implications. However, it has been reported that ACS is the most costly disease than any diagnostic group (Benjamin et al., 2018). Our findings suggest that depression screening and treatment might significantly reduce MACE occurrence, but further evaluation is needed to estimate cost-effectiveness in more detail.

## Supplementary material

For supplementary material accompanying this paper visit https://doi.org/10.1017/S003329171900388X. click here to view supplementary material
