# Long-term cardiac outcomes of depression screening, diagnosis and treatment in patients with acute coronary syndrome: the DEPACS study—ADDENDUM

**DOI:** 10.1017/S0033291720001270

**Published:** 2021-04

**Authors:** Jae-Min Kim, Robert Stewart, Hee-Ju Kang, Seon-Young Kim, Ju-Wan Kim, Hee-Joon Lee, Ju-Yeon Lee, Sung-Wan Kim, Il-Seon Shin, Min-Chul Kim, Hee-Young Shin, Young Joon Hong, Youngkeun Ahn, Myung Ho Jeong, Jin-Sang Yoon

The above mentioned article was published in *Psychological Medicine* with the some conflicts of interest information missing. The correct conflict of interest statement is written below:

## Confilict of Interest

H. Lundbeck A/S provided the study drug.

The authors apologise for this error.
