# Supplementary material for: Long-term cardiac outcomes of depression screening, diagnosis and treatment in patients with acute coronary syndrome: the DEPACS study
Source: Psychol Med. 2020 Jan 7;51(6):964–74. doi: 10.1017/S003329171900388X (PMC8161433; doi:10.1017/S003329171900388X)
Supplement: Supplementary file 1 [file S003329171900388Xsup001.docx]

**Supplementary Material**

**Eligibility Criteria for the K-DEPACS and EsDEPACS participants**

For the K-DEPACS study entry, inclusion criteria were as follows: i) aged 18~85 years; ii) confirmed ACS by investigation (the presence of ST-segment elevation MI was determined by >30 min of continuous chest pain, a new ST-segment elevation ≥2 mm on at least two contiguous electrocardiographic leads, and creatine kinase-MB more than three times normal; the presence of non-ST-segment elevation MI was diagnosed by chest pain and a positive cardiac biochemical marker without new ST-segment elevation; and the presence of unstable angina was determined by chest pain within the preceding 72 h with or without ST-T wave changes or positive cardiac biochemical markers); iii) ability to complete study questionnaires; iv) ability to understand the study objectives and sign informed consent. Exclusion criteria were: i) occurrence of ACS while hospitalized for another reason; ii) ACS developing less than 3 months after a coronary artery bypass graft procedure; iii) uncontrolled hypertension (systolic blood pressure (BP) >180mmHg or diastolic BP >100mmHg); iv) resting heart rate <40/min; v) severe physical illnesses threatening life or interfering with the recovery from ACS; vi) persistent clinically significant laboratory abnormalities in complete blood cell counts, thyroid tests, renal function tests, and liver function tests. For the EsDEPACS study entry, additional inclusion criteria were as follows: i) BDI>10; ii) major or minor depressive disorder according to DSM-IV criteria. Additional exclusion criteria were: i) concomitant use of class I antiarrhythmic medications, reserpine, guanethidine, clonidine, methyldopa, lithium, anticonvulsants, antipsychotics, or antidepressants; ii) history of neuropsychiatric illnesses such as dementia, Parkinson’s disease, brain tumor, psychosis, bipolar disorder, alcoholism, or other substance dependence; iii) pregnancy; iv) participating in other drug trials.
